# Nine complete chloroplast genomes of the *Camellia* genus provide insights into evolutionary relationships and species differentiation

**DOI:** 10.1038/s41598-025-87764-4

**Published:** 2025-03-13

**Authors:** Yanfei Cai, Min Tian, Yingjie Yang, Ziming Shi, Peifei Zhao, Jihua Wang

**Affiliations:** 1 Flower Research Institute of Yunnan Academy of Agricultural Sciences, Kunming, 650000 Yunnan China; 2 Yunnan Flower Technology Innovation Center, Kunming, 650000 Yunnan China

**Keywords:** *Camellia* genus, *Camellia japonica*, Chloroplast genome, Genome structure, SSR molecular marker, Phylogenetic analysis, Sequencing, DNA sequencing, DNA, Phylogenetics

## Abstract

The genus *Camellia,* known for species such as *Camellia japonica*, is of significant agricultural and ecological importance. However, the genetic diversity and evolutionary relationships among *Camellia* species remain insufficiently explored. In this study, we successfully sequenced and assembled the complete chloroplast (cp) genomes of nine *Camellia* accessions, including the species *Camellia petelotii*, and eight varieties of *C. Japonica* (*C. Japonica ‘Massee Lane’*, *C. Japonica ‘L.T.Dees’*, *C. Japonica ‘Songzi’*, *C. Japonica ‘Kagirohi’*, *C. Japonica ‘Sanyuecha’*, *C. Japonica ‘Xiameng Hualin’*, *C. Japonica ‘Xiameng Wenqing’*, and *C. Japonica ‘Xiameng Xiaoxuan’*). These genomes exhibited conserved lengths (~ 156,580–157,002 bp), indicating minimal variation in genome size. They consistently predicted 87 protein-coding genes, although variations were observed in the rRNA and tRNA genes. Structural and evolutionary analyses revealed the highly conserved nature of these cp genomes, with no significant inversions or gene rearrangements detected. Consistent codon usage patterns were observed across these accessions. Five hypervariable regions (*rpsbK*, *psbM*, *ndhJ*, *ndhF*, and *ndhD*) were identified as potential molecular markers for species differentiation. Phylogenetic analysis of 82 accessions from the *Camellia* genus, along with outgroup accessions revealed close genetic relationships among certain *C. japonica* varieties, including *Songzi, Sanyuecha, L.T.Dees, and Kagirohi,* which formed sister groups. *Massee Lane* was located within Sect. *Camellia*. Moreover, *Xiameng Hualin*, *Xiameng Wenqing*, *Xiameng Xiaoxuan*, and *C. petelotii* demonstrated a strong genetic affinity. These findings provide valuable insights into the structural and evolutionary dynamics of *Camellia* cp genomes, contributing to species identification and conservation.

## Introduction

*Camellia*, the largest genus within the Theaceae family, comprises a diverse group of flowering plants with substantial ornamental, economic, and ecological significance^1–3^. Among the *Camellia* species, *Camellia japonica* stands out as one of the most widely cultivated, renowned for its significant floral beauty and ornamental value. Despite its popularity, the genetic diversity and evolutionary relationships within *C. japonica*, as well as among other species in the genus remain insufficiently understood. Currently, this genus is faced with various challenges, such as discrepancies in genetic relationships, difficulties in species identification, and ongoing debates regarding the parentage of numerous *Camelli*a species. While *Camellia* species exhibit limited morphological diversity and have been extensively hybridized to develop new cultivars, they also display trait variability within the same species under different environmental conditions, which complicates morphological classification^4^. Therefore, relying solely on morphological features for species identification is inadequate. Accurate species identification requires a comprehensive phylogenetic analysis using whole-genome sequencing and molecular markers. Chloroplast (cp) genomes, which offer valuable insights into plant evolution, systematics, and phylogenetics, remain underexplored across the diverse varieties of *Camellia.* While previous studies have characterized the cp genomes of several *Camellia* species and provided insights into the phylogenetic relationships within the genus^5–8^, its phylogenetic reconstruction is not incomplete.

Cp genomes in plants are recognized for their highly conserved evolutionary nature and typically adopts a circular, double-stranded quadripartite structure. These genomes comprise a large single-copy (LSC) region, a small single-copy (SSC) region, and a pair of inverted repeat (IR) regions. The two IRs, which are identical in length and oriented in opposite directions, are separated by the LSC and SSC regions^9^. Cp genomes are generally conserved in structure and gene content across plant species. Their relatively simple structure and small genome size, coupled with features such as moderate evolutionary rates, low recombination and variation frequency, maternal inheritance, and independent inheritance patterns^10,11^, make cp genomes highly valuable for applications in molecular marker development, phylogenetic research, and genetic engineering in plant breeding^12^. Comparative cp genomics has therefore emerged as a powerful tool for exploring plant evolutionary relationships, facilitating species identification, and supporting conservation initiatives. Advances in next-generation sequencing technologies have further enabled the complete sequencing of cp genomes, allowing for detailed comparative analyses across species and cultivars. Based on whole-genome resequencing technology, this study focuses on the cp genomes of nine accessions from the *Camellia* genus, including *Camellia petelotii, and* eight varieties of the species *C. japonica* (*C. Japonica* ‘Massee Lane’, *C. Japonica* ‘L.T.Dees’, *C. Japonica* ‘Songzi’, *C. Japonica* ‘Kagirohi’, *C. Japonica* ‘Sanyuecha’, *C. Japonica* ‘Xiameng Hualin’, *C. Japonica* ‘Xiameng Wenqing’, and *C. Japonica* ‘Xiameng Xiaoxuan’). Our primary objectives were uncover structural variations and conserved features, and explore their evolutionary trajectories. Furthermore, we aimed to identify hypervariable regions within cp genomes that can serve as molecular markers for species identification and phylogenetic studies. A phylogenetic tree was constructed using cp genome data from 82 accessions from the *Camellia* genus, with *Zea mays*, *Hordeum vulgare* , *Triticum aestivum* , *Brachypodium distachyon* , *Ficus microcarpa*, four varieties of *Arabidopsis thaliana*, as well as four species of the *Diospyros* genus included as the outgroup. We seek to elucidate the genetic relationships and evolutionary patterns, with a particular focus on* C. japonica* and its closely related species. Our findings contribute valuable insights into the structural and evolutionary dynamics of cp genomes in the *Camellia* genus, laying a foundation for future research on genetic diversity, species identification, and conservation strategies.

## Results

### Complete cp genome overview of the nine *Camellia* accessions

**Fig. 1:**
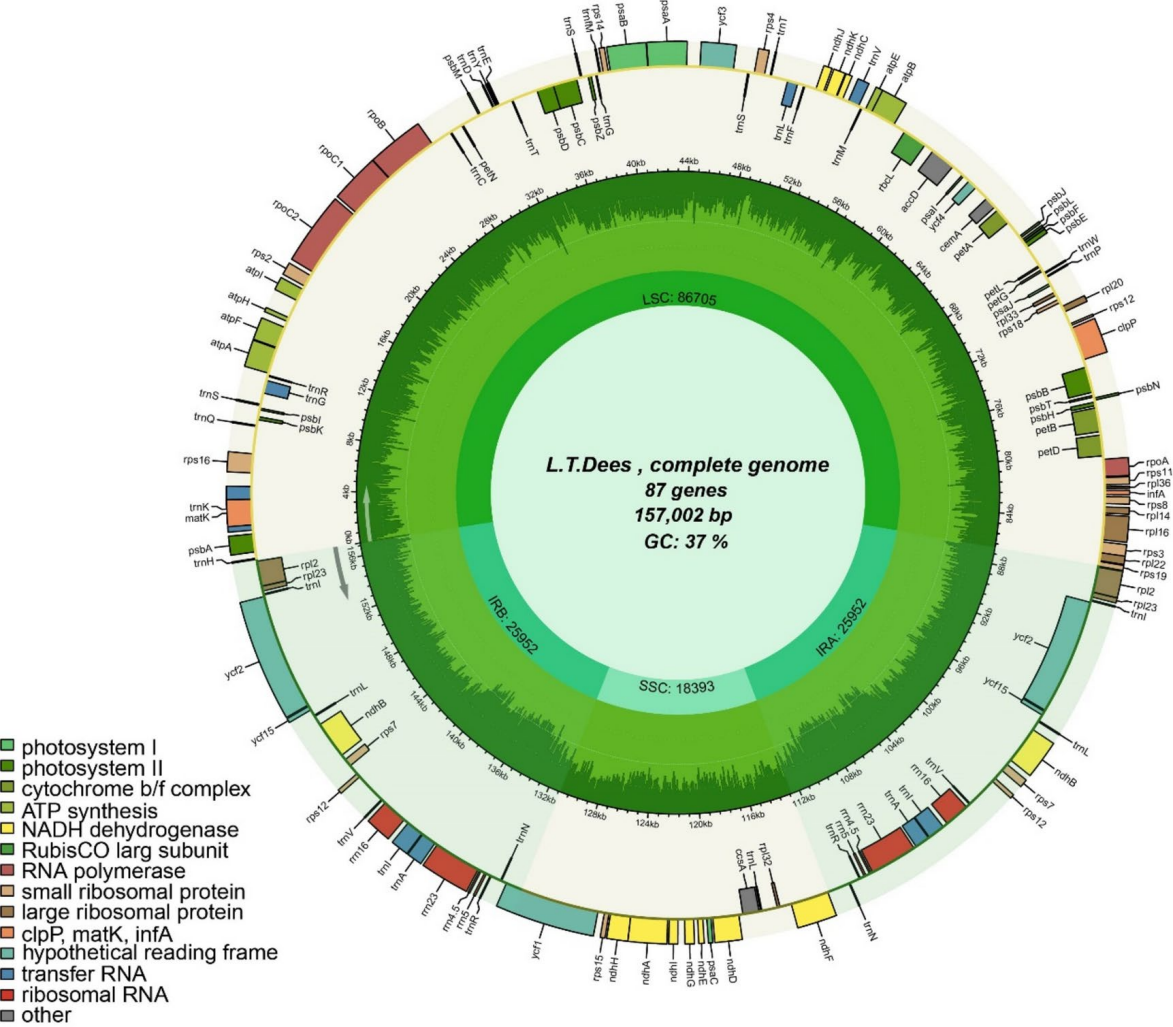
Circular map showing the complete chloroplast (cp) genome of *Camellia japonica* ‘*L.T. Dees’*. This map includes key features of a cp genome, including the large single-copy (LSC) region, small single-copy (SSC) region, and a pair of inverted repeat (IR) regions. The genome is composed of 87 genes, with a total length of 157,002 bp and a GC content of 37%.

We first assembled and annotated the complete cp genomes of the nine *Camellia* accessions using whole genome sequencing. All nine genomes displayed a typical quadripartite structure, consisting of an LSC, an SSC, and two IRs (Fig. 1, Figure S1-8). They were predicted to annotate 87 coding sequences (CDSs) and 8 rRNAs each. Both *Xiameng Hualin* and *C. petelotii* were predicted to annotate 36 tRNAs, while the remaining seven accessions were annotated with 37 tRNAs. The nine cp genomes exhibited minimal length variation, with the genome of *Xiameng Hualin* being the longest at 157,059 bp, and that of *Xiameng Xiaoxuan* being the shortest at 156,580 bp. Moreover, the lengths of the LSC regions, SSC regions and IR regions ranged from 86,589 bp to 86,761 bp, 18,140 bp to 18,394 bp, and 25,953 bp to 26,134 bp, respectively, with the total GC content varying between 37.28 and 37.31%. Among these regions, the IR regions accounted for the highest GC content (42.96–43.03%), followed by the LSC regions (30.54–35.31%), while the SSC regions had the lowest GC content (30.54–30.6%) (Table S1). The higher GC content in the IR regions suggests that the Camellia cp genomes were stable and highly conserved.

### Codon usage analysis

**Fig. 2:**
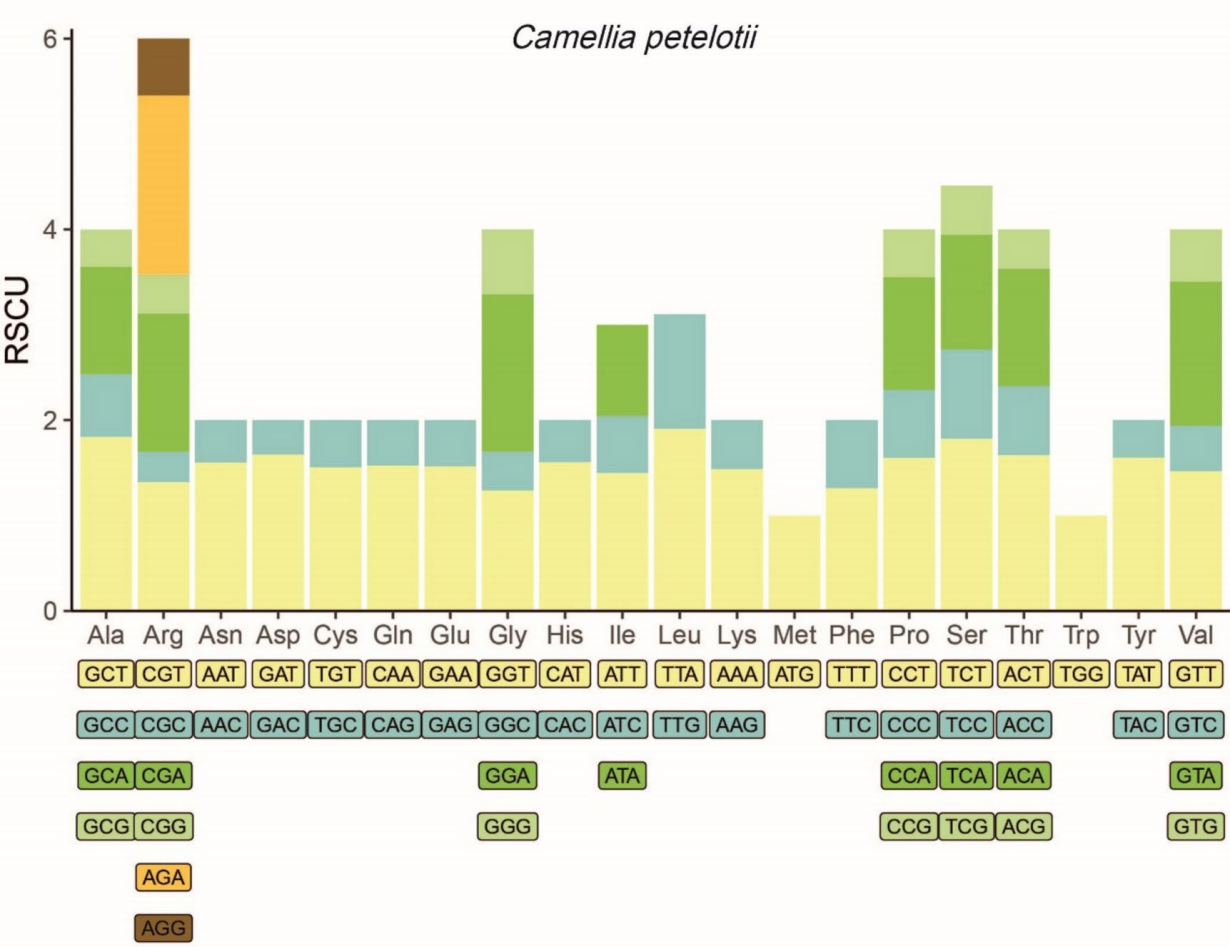
Codon usage analysis of coding sequences (CDSs) from the cp genomes of *C. petelotii*.

Codon usage analysis was conducted on the CDSs from the complete cp genomes of the nine *Camellia* accessions. A total of 20 amino acids were encoded, each with 1–6 synonymous codons in the nine accessions. Differences were observed in the codons encoding leucine (Leu). Specifically, *Kagirohi*, *L.T.Dees*, *Sanyuecha*, *Songzi*, and *Xiameng Wenqing* each used four synonymous codons for Leu (CTT, CTC, CTA, CTG), while *C. petelotii*, *Massee Lane*, *Xiameng Hualin*, and *Xiameng Xiaoxuan* employed only two synonymous codons for Leu (TTA, TTG). The majority of amino acids exhibited a pronounced codon preference, with the notable exception of methionine (Met) and tryptophan (Trp). Each accession contained 29 codons with an RSCU value greater than 1. Moreover, most of these codons ended in A/T bases, indicating a strong preference for A/T usage (Fig. 2, Figure S9, Table S2). This A/T preference represents a common phenomenon observed in the cp genomes of higher plants. These findings highlight the high degree of evolutionary conservation in the cp genomes of the nine *Camellia* accessions.

### Analyses of simple sequence repeats (SSRs) and dispersed repeats

Sequence analysis of the cp genomes of nine Camellia accessions revealed the distribution of dinucleotides to hexanucleotides. Specifically, a total of 16 SSRs were detected in each of the following accessions: *Sanyuecha* (J100), *L.T.Dees* (J49), *Massee Lane* (J51), *Xiameng Hualin* (J105), *Xiameng Wenqing* (J106) and *Xiameng Xiaoxuan* (J108). In contrast, 19 SSRs were identified in *C. petelotii (Cpet)*. Moreover, *Kagirohi* (J94) and *Songzi* (J72) contained 17, and 15 SSRs, respectively. The distribution of SSRs was featured by significant heterogeneity, with the highest frequency observed in the LSC region, while the lowest detected in the SSC region. Tetranucleotide repeats was the most prevalent while pentanucleotide repeats were absent. AT/TA repeats were more frequently observed across the genomes. Three SSR types were exclusively identified in *C. petelotii* (Cept): ATT (12 bp), CTTTTT (18 bp), and AAAAAG (18 bp) (Table 1).

**Table 1:** Simple sequence repeats (SSR) of cp genomes of the nine *Camellia* accessions.

SSR type	Length/bp									SSR	Annotation	Region
	Cept	J100	J49	J51	J94	J105	J106	J108	J72			
p4	12	12	12	12	12	12	12	12	12	(AGAT)3	rps16	LSC
p4	12	12	12	12	12	12	12	12	12	(GTCT)3	atpA	LSC
p2	10	10	10	10	10	10	10	10	10	(AT)5	rpoC2	LSC
p4	12	12	12	12	12	12	12	12	12	(TCTT)3	psbD	LSC
p4	12	12	12	12	12	12	12	12	12	(TTTC)3	ycf3	LSC
p4	12	12	12	12	12	12	12	12	12	(AAAT)3	ycf4	LSC
p4		12	12		12	12		12		(AAAT)3	psaJ	LSC
p3	12	12	12	12	12	12	12	12	12	(TTC)4	rpl33	LSC
p3	12	–	–	–	–	–	–	–	–	(ATT)4	psbB	LSC
p2	10	–	–	10	10	–	10	–	–	(AT)5	rpl16	LSC
p2	10	10	10	10	12	10	10	10	10	(TA)5	rpl2	IRA/B
p4	12	12	12	12	12	12	12	12	12	(TCTA)3	ycf2	IRA/B
p4	12	12	12	12	12	12	12	12	12	(CCCT)3	ndhF	SSC
p6	18	–	–	–	–	–	–	–	–	(CTTTTT)3	rps7	IRA/B
p4	12	12	12	12	12	12	12	12	12	(GAAA)3	ndhD	SSC
p4	12	12	12	12	12	12	12	12	12	(AATA)3	psaC	SSC
p6	18	–	–	–	–	–	–	–	–	(AAAAAG)3	ycf1	SSC
p4	12	12	12	12	12	12	12	12	12	(GAGG)3	rps7	IRA/B
p4	12	12	12	12	12	12	12	12	12	(ATAG)3	ycf2	IRA/B
p2	10	10	10	10	10	10	10	10	10	(AT)5	rpl2	IRB
Total	19	16	16	16	17	16	16	16	15			

The SSR types p2, p3, p4 and p6 represent 2-, 3-, 4- and 6-nucleotide repeat units, respectively. The numbers in the SSR column correspond to the number of sequence repeats. Cept: *Camellia petelotii*; J100: *Sanyuecha*; J49: *L.T.Dees*; J51: *Massee Lane*;J94: *Kagirohi*; J105: *Xiameng Hualin*; J106: *Xiameng Wenqing*; J108: *Xiameng Xiaoxuan*; J72: *Songzi*.

**Fig. 3:**
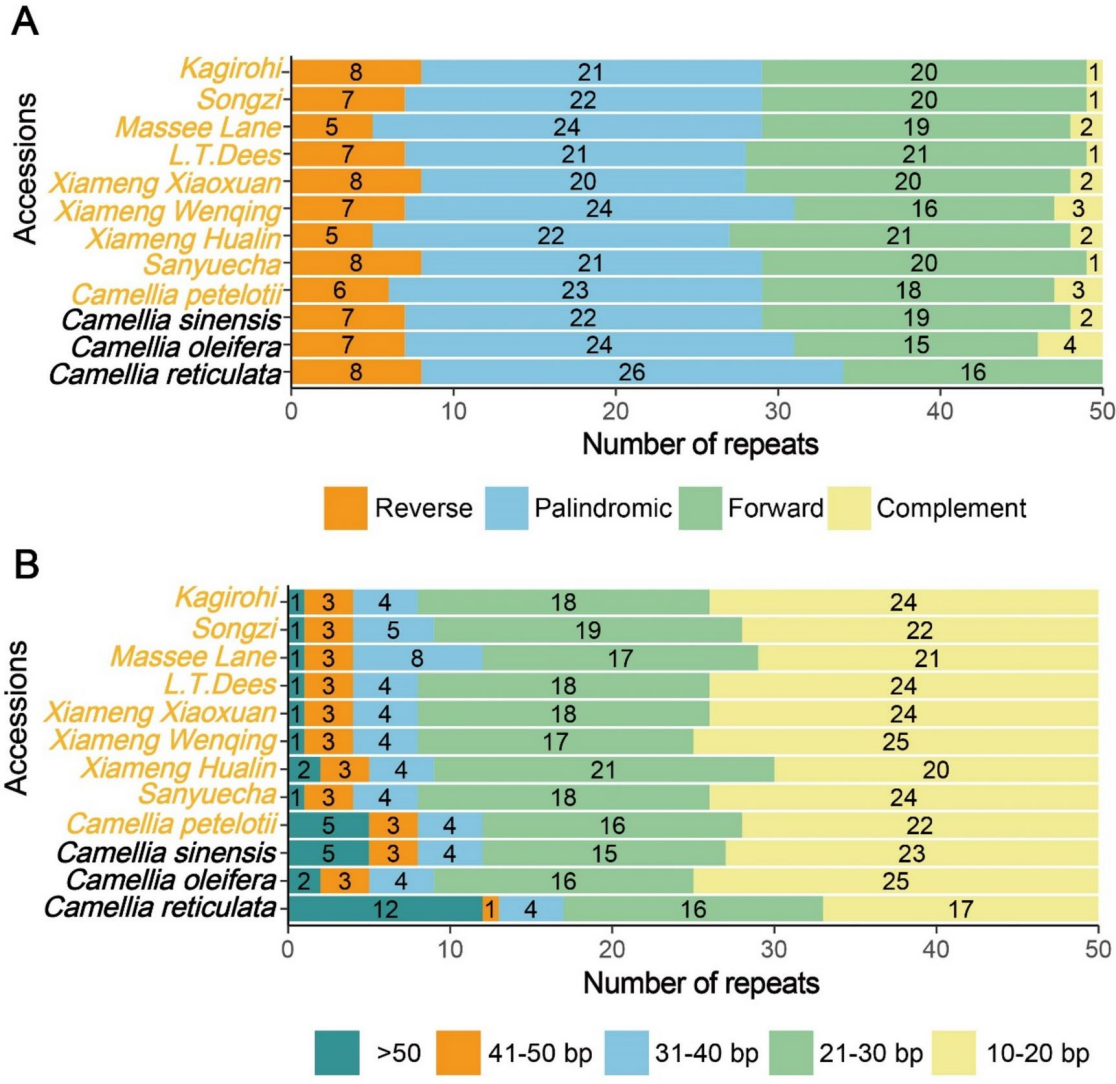
Dispersed repeat analysis. (**A**) Number of different types of dispersed repeats; (**B**) Number of dispersed repeats of different lengths.

Dispersed repeat analysis was conducted across the nine *Camellia* accessions and three reference genomes: *Camellia reticulata*, *Camellia oleifera*, and *Camellia sinensis* cv. *Wuyi Narcissus*. A total of 600 dispersed repeats were identified, encompassing all four types, namely forward, reverse, palindromic and complement. Among them, 270 were palindromic sequences, accounting for 45% of all detected dispersed repeats (Fig. 3A). Length-based categorization revealed five distinct groups: 10–20 bp, 21–30 bp, 31–40 bp, 41–50 bp, and > 50 bp. The 10–20 bp category was the most prevalent, followed by 21–30 bp, with > 50 bp sequences being the least frequent. *C. petelotii* exhibited a significantly higher number of 10–20 bp repeats compared with other accessions. In contrast, *C. reticulata* displayed a markedly lower number of 41–50 bp repeat sequences, but a significantly higher number of > 50 bp sequences than the other *Camellia* accessions (Fig. 3B).

### Collinearity analysis

**Fig. 4:**
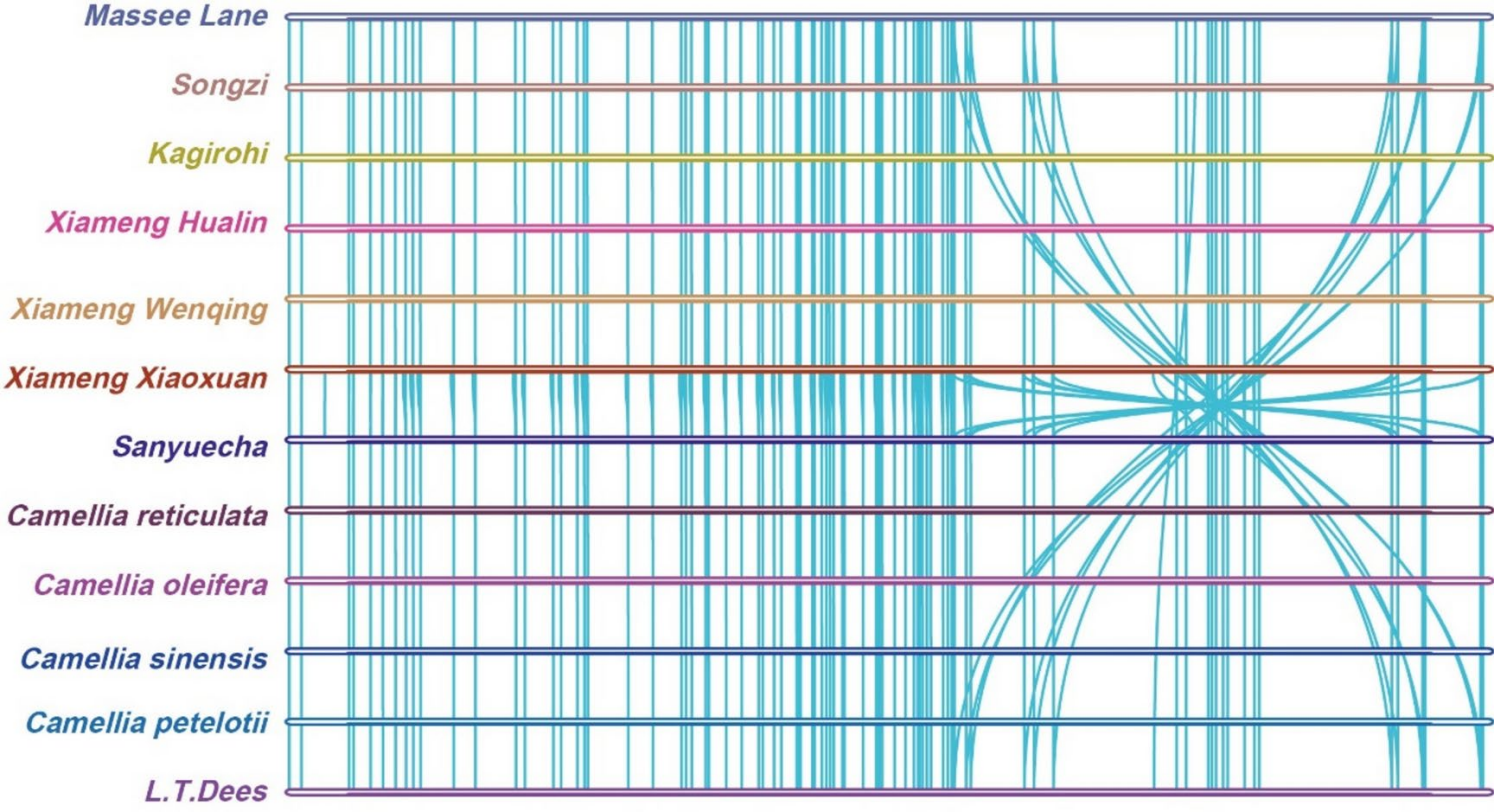
Collinearity analysis of cp genomes.

Collinearity analysis was conducted using the MCScanX tool within the Tbtools software on the nine *Camellia* genomes and three reference genomes. The results revealed a high degree of similarity among the cp genomes, with no large-scale inversions or rearrangements observed in their genetic structure (Fig. 4). Such a collinear relationship among genome structures and gene sequences demonstrate substantial homologous similarity across these cp genomes.

### Expansion and contraction of the IR region

**Fig. 5:**
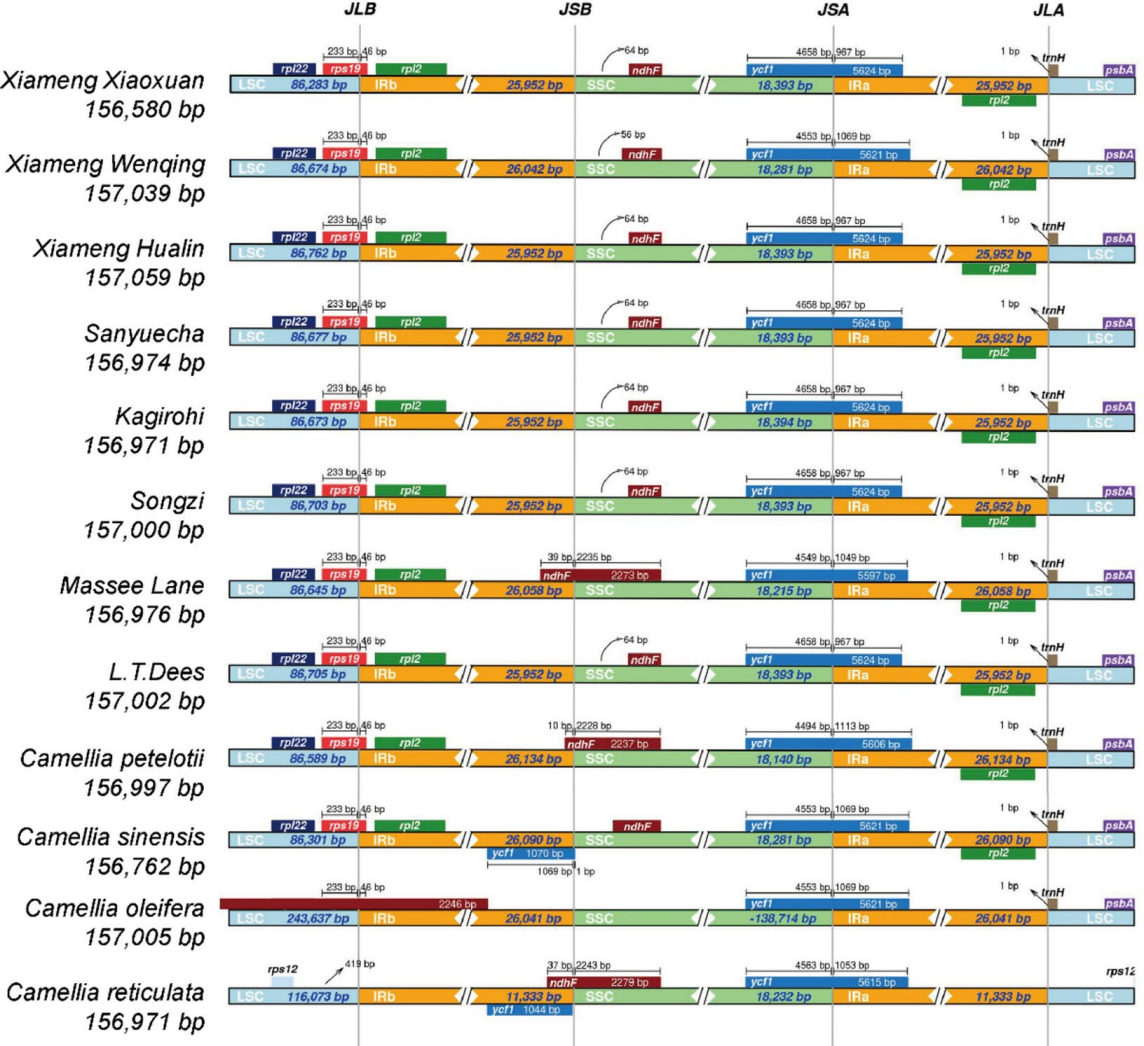
Comparative analysis of the expansion and contraction of IR regions in the cp genomes across the nine *Camellia* accessions, incorporating three reference genomes. JLB: junction between LSC and IRb; JSB: junction between IRb and SSC; JSA: junction between SSC and IRa; JLA: junction between IRa and LSC.

To investigate structural variations of *Camellia* cp genomes, a comparative analysis was conducted to evaluate the boundaries between the IR, LSC, and SSC regions of the nine *Camellia* accessions, combining the three reference genomes. The results revealed that the cp genomes were generally conserved, although some variations in the genome boundaries were observed (Fig. 5). Notably, *C. petelotii* exhibited a longer IR region and a shorter SSC region compared with other accessions, suggesting a potential expansion of the IR region during its evolutionary history. Except for the reference species *C. reticulata* and *C. oleifera*, the *rps19* gene in the remaining 10 species spanned both the LSC and IRb regions, with an extension of 46 bp into the IRb. The *rpl2* gene was consistently located within the IR region across all accessions. The *ndhF* gene in *Massee lane*, *C. petelotii*, and* C. reticulata* spanned both the IRb and SSC regions, whereas in the other nine *Camellia* accessions, the *ndhF* gene was confined to the SSC region. The *ycf1* gene in all the 12 genomes spanned both the SSC and IRa regions, with the portion located within the IRa region showing little variation (ranging from 967 to 1,113 bp). With the exception of *C. reticulata*, the *trnH* gene in the remaining 11 accessions was located in the LSC region, with a uniform expansion of 1 bp towards the *trnH* gene from the IR region.

### Analyses of nucleotide polymorphism (Pi) and selective pressure

**Fig. 6:**
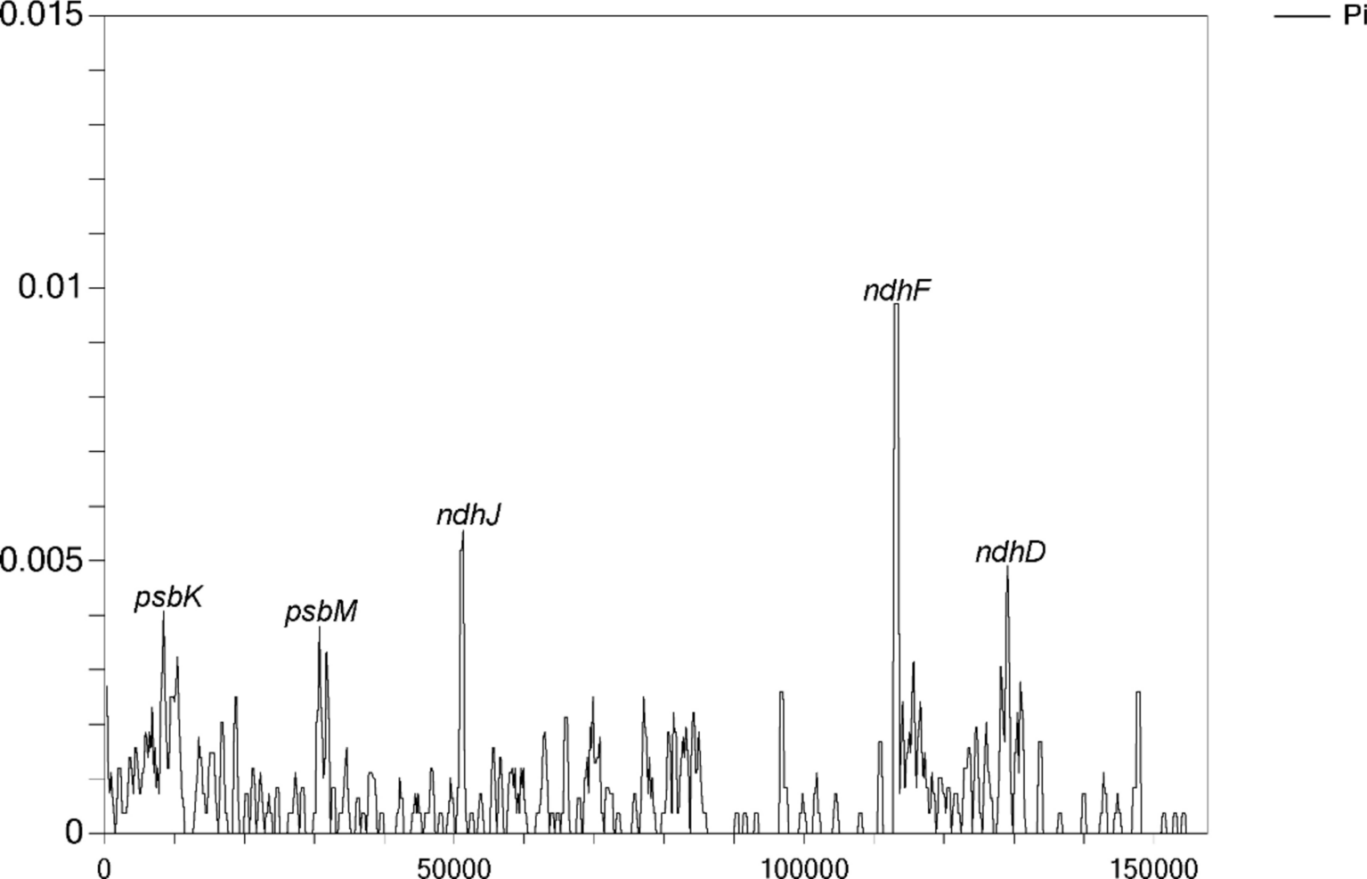
Nucleotide polymorphism (Pi) analysis of cp genome sequences in the nine *Camellia* accessions. Five sites with high nucleotide diversity were identified: *rpsbK*, *psbM*, *ndhJ*, *ndhF* and *ndhD*.

To delve deeper into the variations in cp genome sequences across the nine *Camellia* accessions, nucleotide Pi analysis was conducted (Fig. 6). The results showed high sequence similarity, with Pi values ranging from 0 to 0.01. Five sites with high nucleotide diversity were identified: *rpsbK*, *psbM*, *ndhJ*, *ndhF* and *ndhD*. Among them, *rpsbK*, *psbM* and *ndhJ* were situated in the LSC region, while ndhF and ndhD were located in the SSC region.

**Fig. 7:**
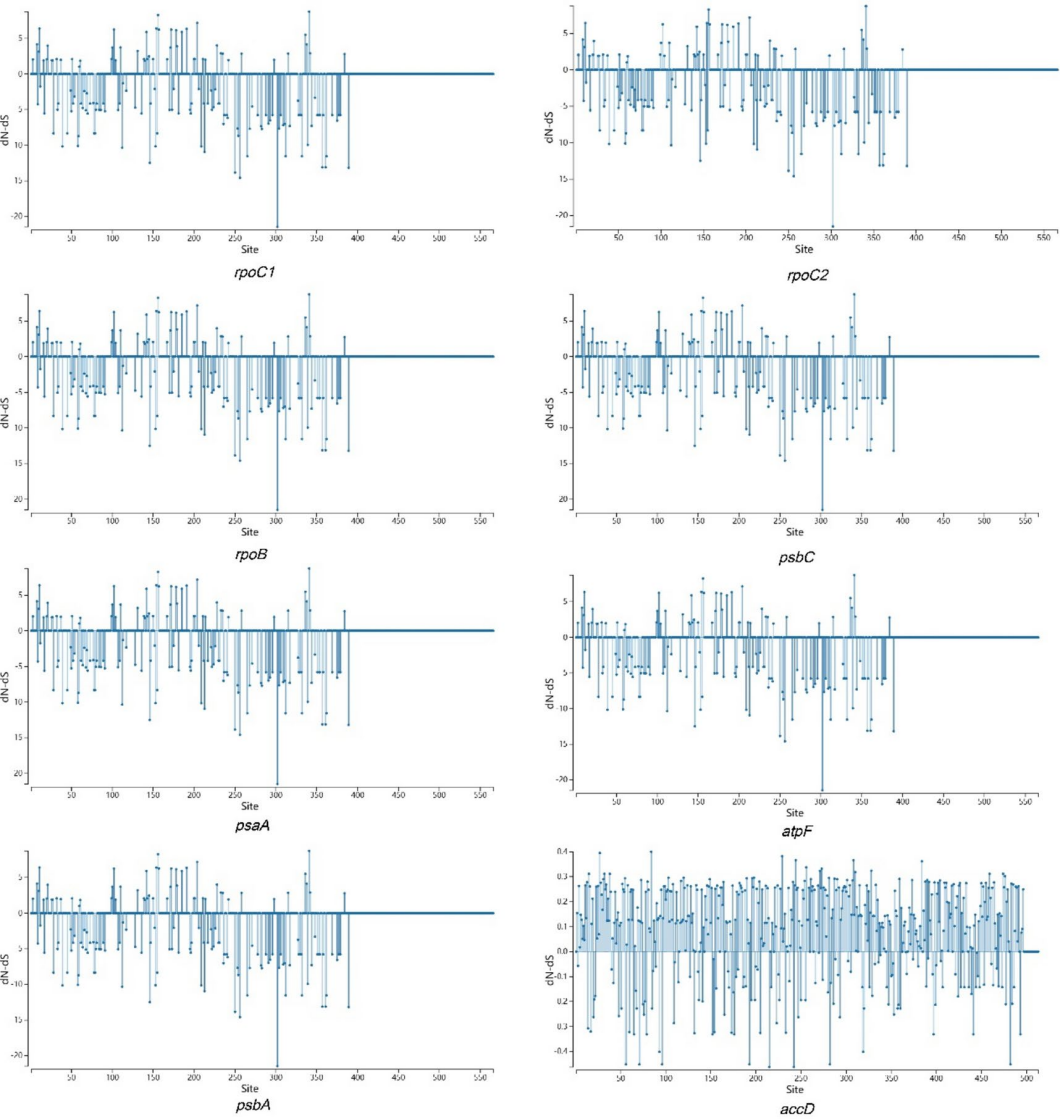
Selective pressure analysis of eight protein-coding genes in the cp genomes of the nine *Camellia* accessions.

The evolutionary rates of protein-coding genes in the cp genomes of the nine *Camellia* accessions were analyzed based on the ratio of non-synonymous substitution rate (dN) to synonymous substitution rate (dS). Ribosomal genes (*rpoC1*, *rpoC2*, and *rpoB*), photosynthesis-related genes (*psbC*, *psaA*, *atpF*, and *psbA*), and the fatty acid synthesis gene *accD*, were selected for analysis. Using the pyphy software, ka-Ks values for these genes within the cp genomes were visually represented. We found that the *accD* gene displayed a notably higher number of sites with dN/dS > 0. This underscores the exceptionally high evolutionary rate of the *accD* gene and further suggests that the *accD*-encoded protein may be under positive selective pressure. Moreover, the results revealed a significant increase in the number of sites with dN/dS < 0 for the CDSs of the remaining genes, indicating of purifying selection (Fig. 7).

### Phylogenetic analysis

**Fig. 8:**
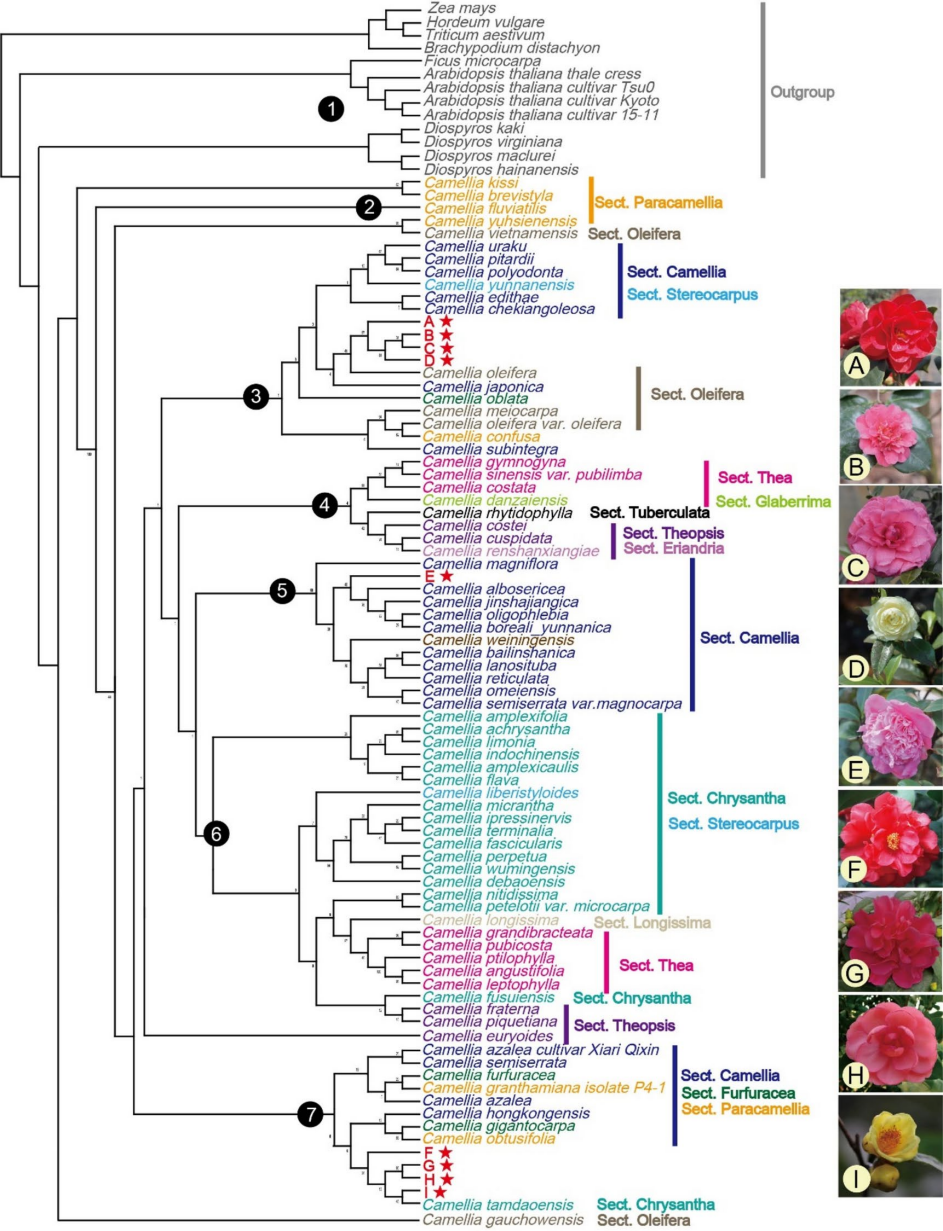
Phylogenetic tree encompassing 82 accessions from the *Camellia* genus, along with 13 outgroup accessions. A: *Songzi*; B: *Sanyuecha*; C: *L.T.Dees*; D: *Kagirohi*; E: Massee Lane;F: *Xiameng Wenqing*; G: *Xiameng Hualin*; H: *Xiameng Xiaoxuan*; I: *Camellia petelotii*.

To clarify the phylogenetic relationship of the *Camellia* genus, a phylogenetic tree was constructed using the cp genomes of 82 accessions from the genus, with *Zea mays*, *Hordeum vulgare* , *Triticum aestivum* , *Brachypodium distachyon* , *Ficus microcarpa*, four varieties of *A. thaliana*, as well as four species of the *Diospyros* genus included as the outgroup. Seven distinct clusters were identified. Four *C. japonica* varieties, namely *Songzi, Sanyuecha, L.T.Dees, and Kagirohi*, clustered with *C. Oleifera* within the Sect. *Oleifera* branch, In contrast, *Massee Lane* was classified under the Sect. *Camellia* branch. For *Xiameng Hualin, Xiameng Wenqing, Xiameng Xiaoxuan,* and *Camellia petelotii*, they grouped together in a clade closed to *Camellia tamdaoensis* within the Sect. *Chrysantha* branch. Additionally, a strong phylogenetic affinity was observed between Sect. *Paracamellia* and Sect*. Oleifera*. Moreover, *Camellia Yunnanensis* and *Camellia liberistyloides* within the Sect. *Stereocarpus* branch were found to be clustered with Sect. *Camellia* and Sect. *Chrysantha*, respectively (Fig. 8).

## Materials and methods

### Sample collection, DNA extraction and sequencing

Plants of the nine *Camellia* accessions were cultivated at the International Flower Technology Innovation Center, Kunming, China. Fresh, healthy leaf samples were collected and immediately frozen in liquid nitrogen on February 12th, 2022. The plant materials used in this study are common species and not classified as endangered. Sampling was conducted in strict accordance with institutional, national, and international regulations. No specific permits were required for the collection of these materials. Species identification was performed by Wenzhong Huang, Huang-Wenzhong Camellia Planting Co., Ltd., Yunnan Province. All plant materials are registered in the Database of International Camellia Register (https://camellia.iflora.cn/). Leaf samples can be obtained upon reasonable request from the International Flower Technology Innovation Center, where the plants are actively growing.

Total genomic DNA was extracted from the samples using cetyltrimethylammonium bromide following a modified protocol^13^. The quality and concentration of the extracted DNA products were assessed using 1% agarose gel electrophoresis and spectrophotometry. After quality assessment, the DNA was fragmented by ultrasonic treatment, and 270 bp DNA fragments were selected for polymerase chain reaction (PCR) amplification. The PCR products were purified by removing the primer dimers, and the resulting fragments were used to construct the library. The prepared library was sequenced on the Illumina platform (Illumina, USA), generating 150 bp paired-end (PE150) reads.

### Cp genome assembly and annotation

Fastp was used to filter out low-quality sequences from raw reads. For each accession, approximately 6 GB of clean data were successfully obtained. The clean data were assembled using the GetOrganelle software^14^. Leveraging the c p genome sequences of *C. reticulate* (KJ806278)*, C. oleifera* (OP953554), and *C. sinensis cv. Wuyi Narcissus* (MT612435) as reference sequences from the National Center for Biotechnology Information (NCBI), we predicted and annotated the cp genome sequences of the nine *Camellia* accessions with Dual Organellar Genome Annotator (DOGMA)^15^ and CPGAVAS2^16^. Following the prediction of CDS, rRNA, and tRNA of the cp genomes, we visualized the complete cp genomes of the nine *Camellia* accessions using Chloroplot^17^.

### Analysis of codon preference and collinearity

Gffread was used to extract CDSs. The codonW software^18^ was utilized to carry out statistical analysis on the codon preference and to calculate RSCU. The results were visualized with stacked bar charts using the R software (version 4.3.1). For collinearity visualization of the cp genomes, MCScanX^19^ within the Tbtools software was used.

### Analysis of cp genome features

The repeat sequences in the cpDNA of the nine *Camellia* accessions, including forward, reverse, palindromic and complement repeats were identified using REPtuter^20^. The parameters for repeat identification were set to a maximum of 50 repeats, with a minimum repeat length of eight nucleotides. SSRs were detected using MISA^21^ with the minimum repeat numbers set at 10, 5, 4, 3, 3, and 3 for mononucleotide, dinucleotide, trinucleotide, tetranucleotide, pentanucleotide, and hexanucleotide, respectively. The minimum distance between two SSRs was set to 100 bp. Based on the annotated GeneBank files, the local IRscope software^22^ was used to visualize the boundaries of the IR region and to observe the expansion and contraction of the IR boundary in the cp genome.

### Pi and selective pressure analyses

Nucleotide polymorphisms of the complete cp genome sequence of the nine *Camellia* accessions were calculated using DnaSP software^23^. The parameters were set as follows: step size = 400 bp, and window length = 600 bp. The Ka/Ks values for each gene were computed using KaKs Calculator software^24^) and the MLWL method.

### Phylogenetic analysis

Gblocks was applied to extract the conserved protein sequences from the cp genomes of 82 accessions of the *Camellia* genus, which were obtained from the NCBI database, with *Zea mays*, *Hordeum vulgare*, *Triticum aestivum*, *Brachypodium distachyon*, *Ficus microcarpa*, and four varieties of *A. thaliana*, as well as four species of *Diospyros* included as the outgroup. Orthofinder was then used to identify sequences of single-copy homologous genes. Sequence alignment was conducted by Muscle. Subsequently, an optimal model was predicted using ProtTest software (v.3.4). Ultimately, the RAxML software^25^ was used to construct a phylogenetic tree based on the JTT + I + G + F model (Bootstrap = 1000). The resulting evolutionary tree was visualized using MEGA (v 11.0.11).

## Discussion

This study sequenced and assembled the complete cp genomes of nine *Camellia* accessions, including *Massee Lane, L.T.Dees, Songzi, Kagirohi, Sanyuecha, Xiamen Hualin, Xiamen Wenqing, Xiamen Xiaoxuan,* and *Camellia petelotii*. Furthermore, annotation of these genomes revealed that the cp genomes of the nine accessions exhibited a typical quadripartite structure^26^. The cp genome lengths of the nine *Camellia* accessions ranged from 156 to 157 kb, consistent with the reported lengths of cp genomes within the *Camellia* genus in previous studies^1,27,28^. After thorough comparison, we found that the primary factor contributing to the variation in the lengths of the cp genomes was in the differences in the LSC region, aligning with findings from a previous study^1^. The GC content of genomes serves as a crucial metric for assessing phylogenetic relationships and sequence stability across species^29^. In the cp genomes of the nine *Camellia* accessions, the average GC content was found to be highest in the two IR regions. This suggests that the IR region may be the most conserved segment of the cp genomes, likely due to the presence of rRNA genes in the region. In this study, the cp genomes of the nine *Camellia* accessions were annotated, identifying 87 protein-coding genes and 8 rRNA genes. No rearrangements or deletions were observed. Notably, in many angiosperm lineages, genes such as *infA* and *rps16* are frequently lost from the cp genome^11,30^, but these genes were retained in the cp genomes of our study.

Repetitive sequences, which carry significant genetic information and are closely associated with sequence variation, tend to be more abundant in species with greater evolutionary complexity^31,32^. In comparison to the Gramineae (132 bp) and Leguminosae (287 bp) families^7^, the repeat sequences within the cp genomes of the nine *Camellia* accessions exhibited a comparatively shorter length (ranging from 10 to 50 bp), with very few sequences exceeding 50 bp. Collectively, these observations suggest that the evolution of cp genomes in the *Camellia* genus may be relatively slow and conservative, which is consistent with a previous report^6^. The contraction and expansion of the IR region significantly influences the length of cp genome^33^, and play crucial roles in cp evolution^34^. In this study, genes located near the IR boundary (*rps19*, *rpl2*, *ndhF*, *ycf1*, and *trnH*) were analyzed. The results revealed that in *Massee Lane*, *C. petelotii*, and *C. reticulata*, the *ndhF* gene spanned both the IRb and SSC regions. These three accessions exhibited position shifts of the *ndhF* gene, as well as length variation, while the other nine accessions showed minimal differences in the distance between the *ndhF* gene and the IR boundary. We propose that the IR/LSC boundary was more conserved than the IR/SSC boundary, which holds significant implications for the study of IR region boundaries, offering valuable insights for phylogenetic analysis and species identification.

SSR analysis identified five types of nucleotide repeats in the cp genomes of the nine *Camellia* accessions. Notably, no pentanucleotide repeats were detected, and hexanucleotide repeats were only found in *C. petelotii*. The remaining four repeat units were consistently observed across all nine accessions. This finding aligned with previous research, which indicates the rare occurrence of pentanucleotide and hexanucleotide repeats within cp genomes^35^. A/T nucleotide repeats are a common feature in most angiosperms^36^. In this study, the most prevalent SSRs were mononucleotide repeats, all consisting of A/T, with a notable A/T base preference also observed in di- to hexanucleotide repeats. Codon usage analysis further revealed that nearly all codons with an RSCU value > 1 terminated with A/T. This suggests that the A/T preference is a crucial evolutionary characteristics of the cp genome. The high proportion of A/T repeats may be one of the factors contributing to the elevated A/T content observed in cp genomes^37^. Accurate species identification in the *Camellia* genus based on morphological traits is often difficult. However, DNA barcoding techniques using cp genomes have become increasingly effective for species identification in recent years^38^. For example, the rpl16 and psbA-tmH sequences have been shown to reliably identify *Camellia pubipetala*^39^, while rbcL, matK and ycf1 have been used as DNA barcodes for other plant species^40^. In our study, Pi analysis identified five highly variable nucleotide sites: *rpsbK*, *psbM*, *ndhJ*, *ndhF* and *ndhD*. These regions possessed strong potential as DNA barcodes and molecular markers. Furthermore, previous research has demonstrated that the *ndhF* gene in the genus *Rheum* contains three positively selected amino acid sites, which are likely associated with environmental adaptation^41^.

Phylogenetic analysis revealed that *Songzi, Sanyuecha, L.T.Dees, Kagirohi* were closely related to Sect. *Camellia* and Sect. *Oleifera. Massee Lane* was found to be clustered with the Sect. *Camellia* branch, while the other four *Camellia* accessions *Xiameng Hualin, Xiameng Wenqing, Xiameng Xiaoxuan, Camellia petelotii* were classified into the same branch, close to *Camellia tamdaoensis* within the Sect. *Chrysantha* branch. This analysis elucidates the phylogenetic positions of the nine *Camellia* accessions, and provides a theoretical foundation for investigating the genetic relationships among *Camellia* species. The taxonomic position of *C. yunnanensis* has long been a subject of debate. In the classification systems of Hongda Zhang^42^ and Tianlu Min^43^, *C. yunnanensis* has been placed in Sect. *Stereocarpus* and Sect. *Heterogenea*, respectively. Our results showed that *C. yunnanensis* was under Sect. *Stereocarpus*, which is consistent with the classification of Hongda Zhang. It clustered with Sect. *Camellia*, suggesting a potentially close phylogenetic relationships between these sections. Meanwhile, we also discovered that Sect. *Theopsis* clustered with two species within Sect. *Eriandria*, which is consistent with previous research^44,45^. Based on previous findings and the results of the current study, we propose that Sect. *Eriandria* and Sect. *Theopsis* form a monophyletic branch. A significant body of literature has suggested that Sect. *Chrysantha* may represent a paraphyletic or polyphyletic group, raising questions about its validity as a distinct taxonomic unit^8,46^. Li et al. found that species of Sect. *Camellia* are predominantly distributed across two branches^1^. Our findings indicate that Sect. *Chrysantha* and Sect. *Camellia* may not form a monophyletic group. Additionally, Wu et al. reported a close relationship between Sect. *Camellia* and Sect. *Oleifera*^4^. The combination of Sect. *Paracamellia* and Sect. *Oleifera* has long been a contentious issue in the classification of *Camellia*. The Hongda Zhang classification system treats these two groups as distinct entities^42^*.* Jiang et al. have found significant differences in flower traits between *C. Oleifera* and the *C. Breviflora*^47^. Shen et al. have also clearly distinguished *C. Oleifera* and *C. Breviflora* using FTIR technology^48^. Tianlu Min demonstrated that Sect. *Oleifera* should be merged with Sect. *Paracamellia*^49^. The results of this study showed that Sect. *Oleifera* and Sect. *Paracamellia* were closely related. Therefore, we supports the merge of Sect. *Paracamellia* and Sect. *Oleifera*, which is consistent with findings from multiple studies^4,38,50^.

## Supplementary Information

Supplementary Information 1.

Supplementary Information 2.

Supplementary Information 3.

## Data Availability

The datasets generated during and/or analysed during the current study are available from GenBase repository under the project PRJCA030917.
