# Supplementary material for: Nine complete chloroplast genomes of the Camellia genus provide insights into evolutionary relationships and species differentiation
Source: Sci Rep. 2025 Mar 13;15:8783. doi: 10.1038/s41598-025-87764-4 (PMC11906861; doi:10.1038/s41598-025-87764-4)
Supplement: Supplementary file 1 — Supplementary Information 1. [file 41598_2025_87764_MOESM1_ESM.docx]

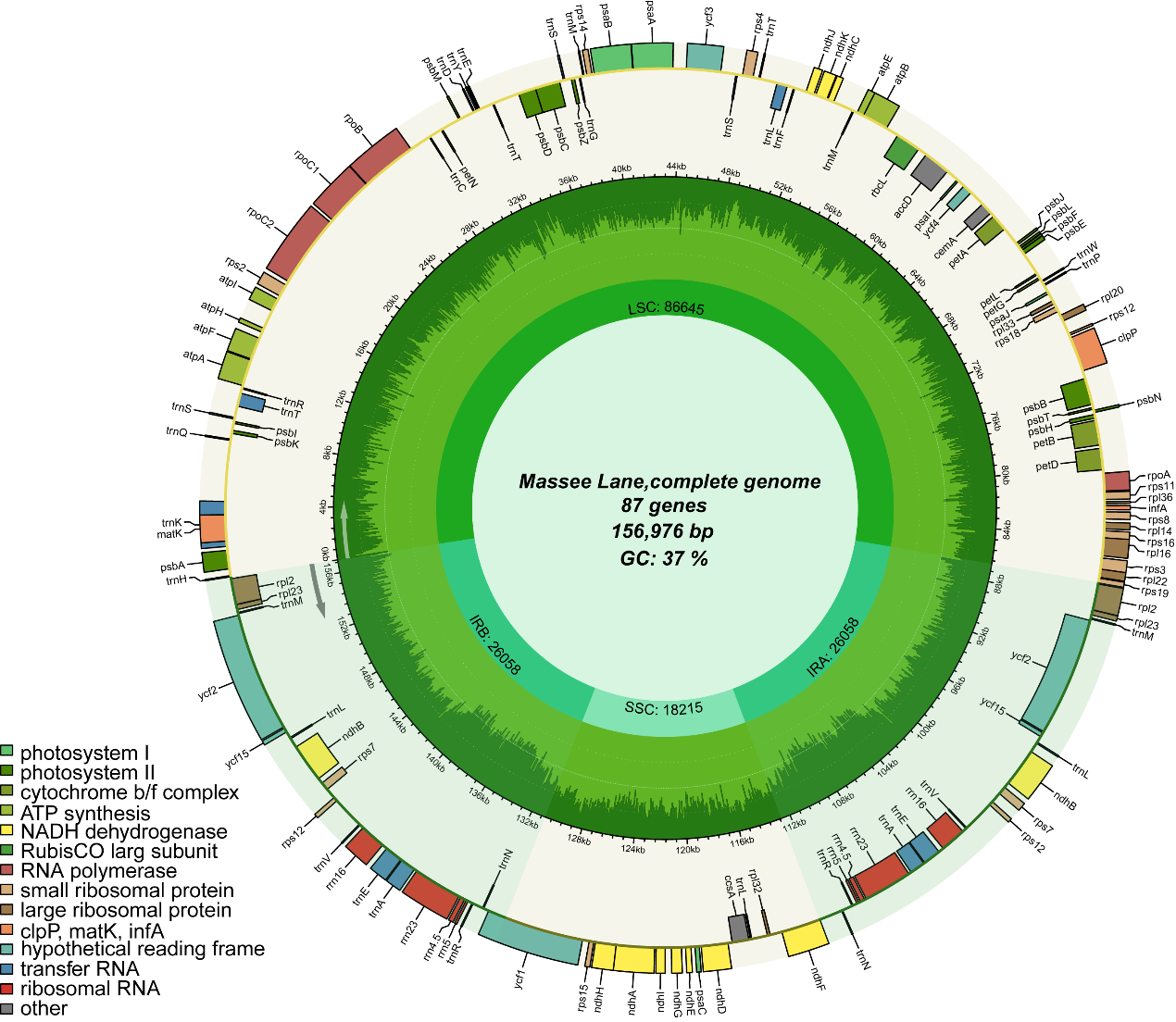


Figure S1 Circular map showing the complete chloroplast (cp) genome of *Camellia japonica* ‘Massee Lane’. This map includes key features of a cp genome, including the large single-copy (LSC) region, small single-copy (SSC) region, and a pair of inverted repeat (IR) regions.


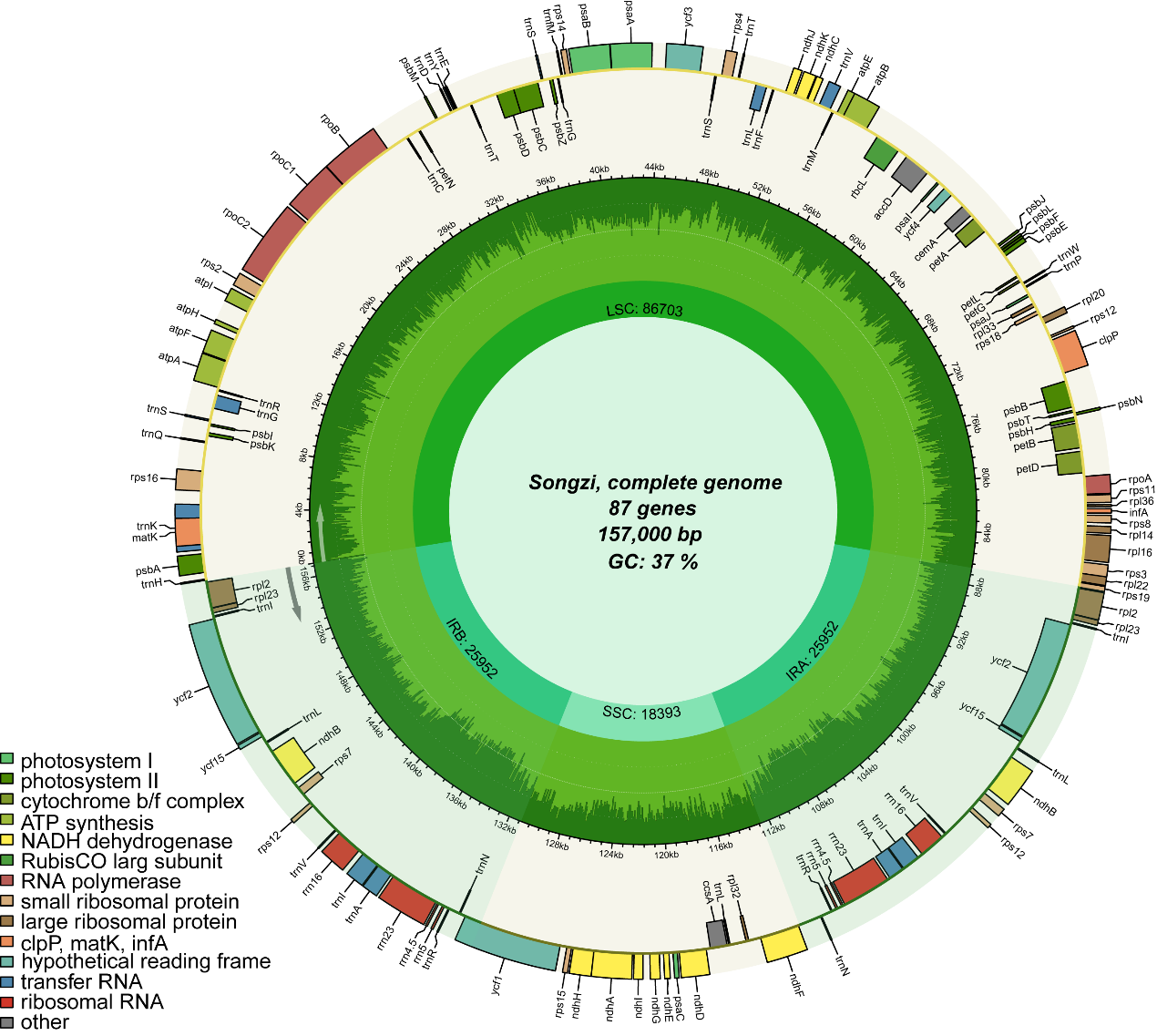


Figure S2 Circular map showing the complete chloroplast (cp) genome of *Camellia japonica* ‘Songzi’. This map includes key features of a cp genome, including the large single-copy (LSC) region, small single-copy (SSC) region, and a pair of inverted repeat (IR) regions.


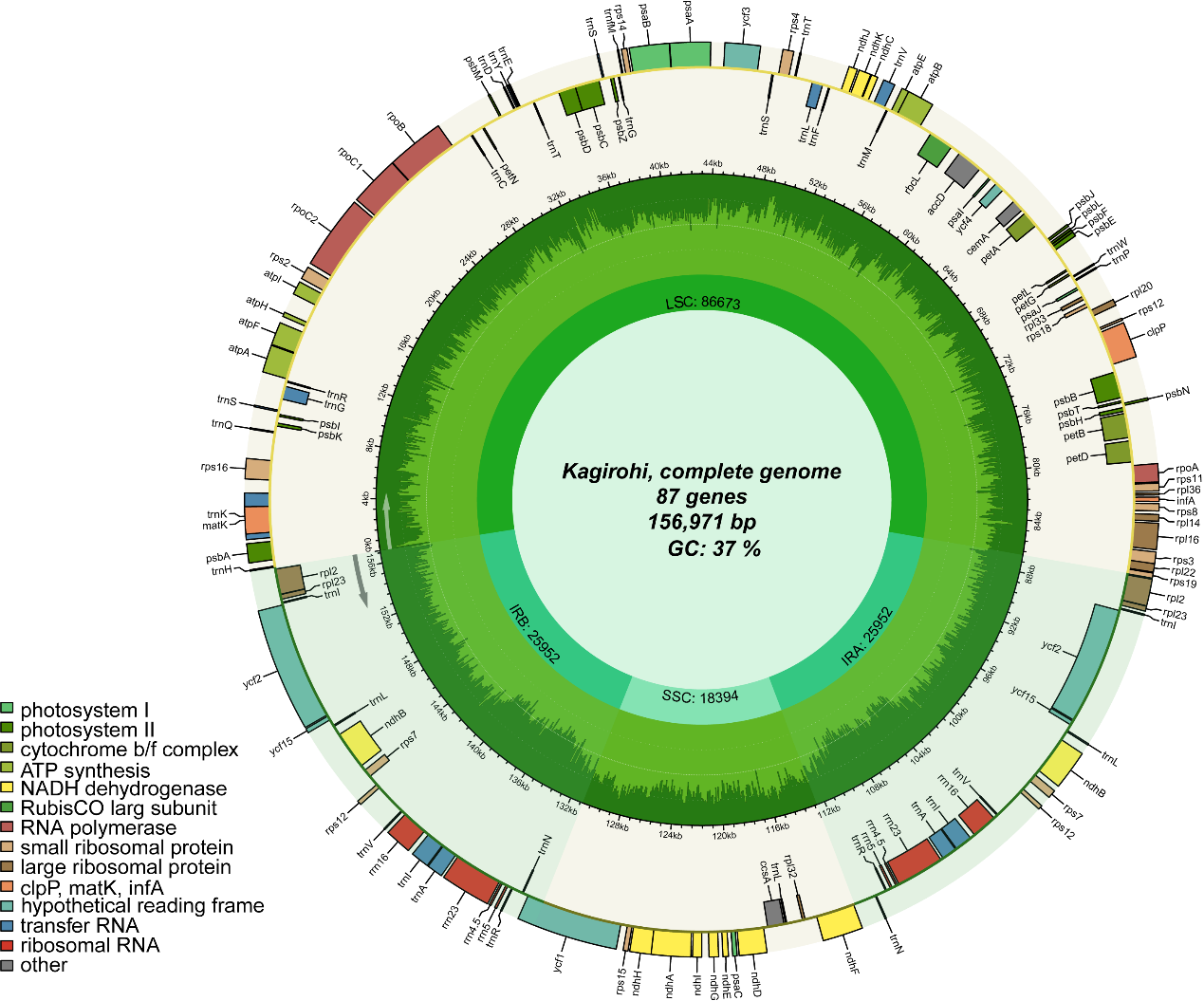


Figure S3 Circular map showing the complete chloroplast (cp) genome of *Camellia japonica* ‘Kagirohi’. This map includes key features of a cp genome, including the large single-copy (LSC) region, small single-copy (SSC) region, and a pair of inverted repeat (IR) regions.


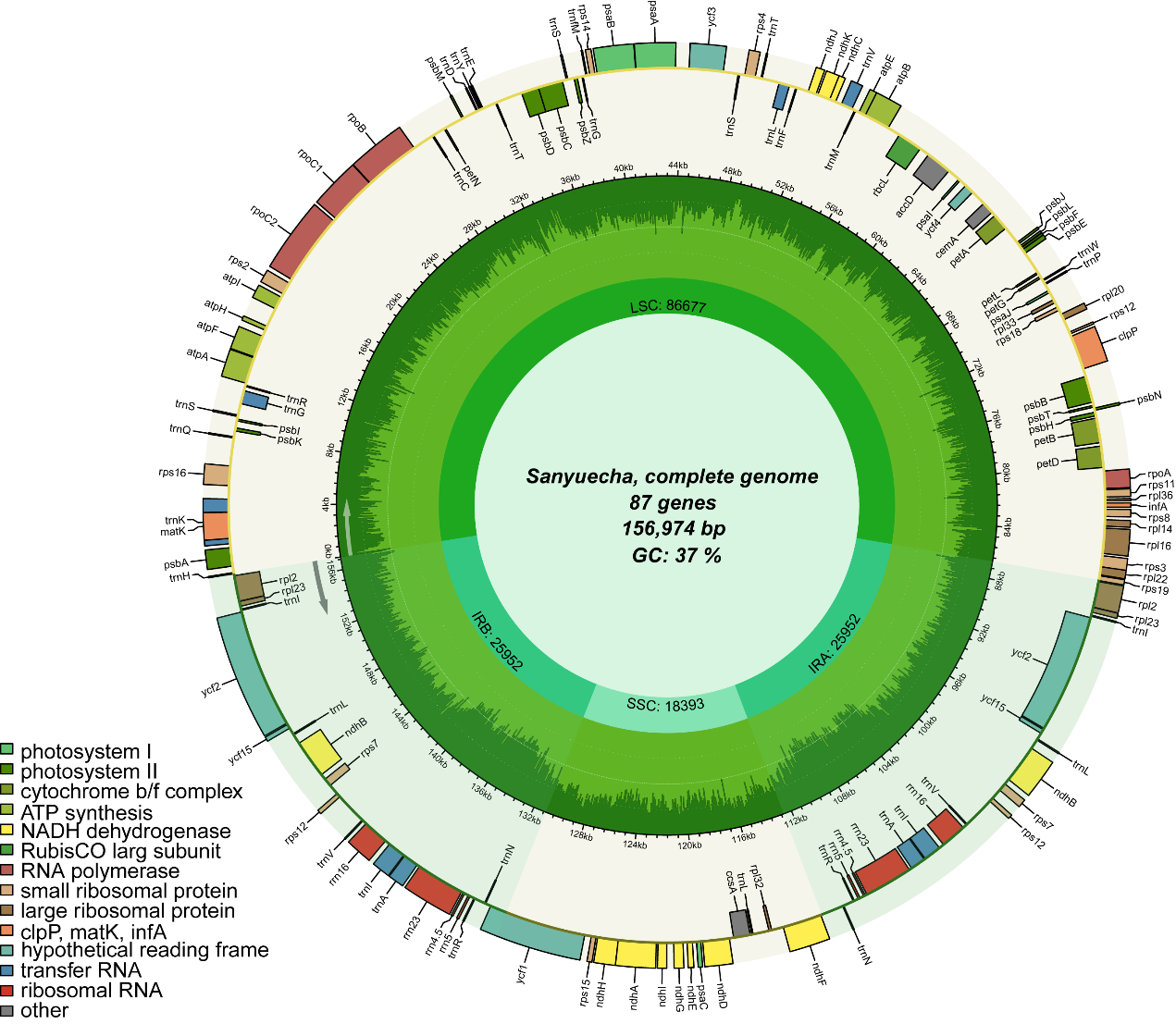


Figure S4 Circular map showing the complete chloroplast (cp) genome of *Camellia japonica* ‘Sanyuecha’. This map includes key features of a cp genome, including the large single-copy (LSC) region, small single-copy (SSC) region, and a pair of inverted repeat (IR) regions.


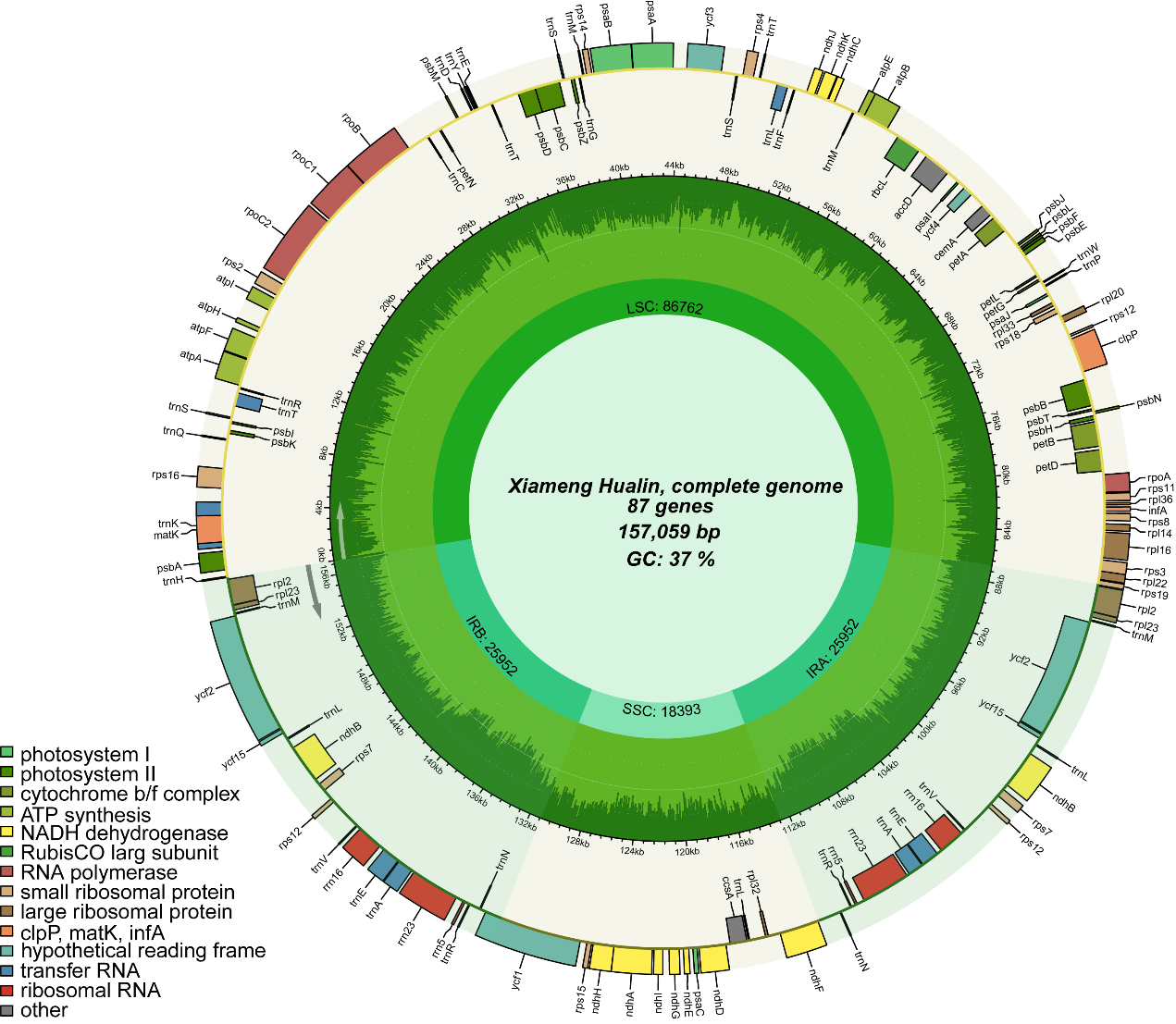


Figure S5 Circular map showing the complete chloroplast (cp) genome of *Camellia japonica* ‘Xiameng Hualin’. This map includes key features of a cp genome, including the large single-copy (LSC) region, small single-copy (SSC) region, and a pair of inverted repeat (IR) regions.


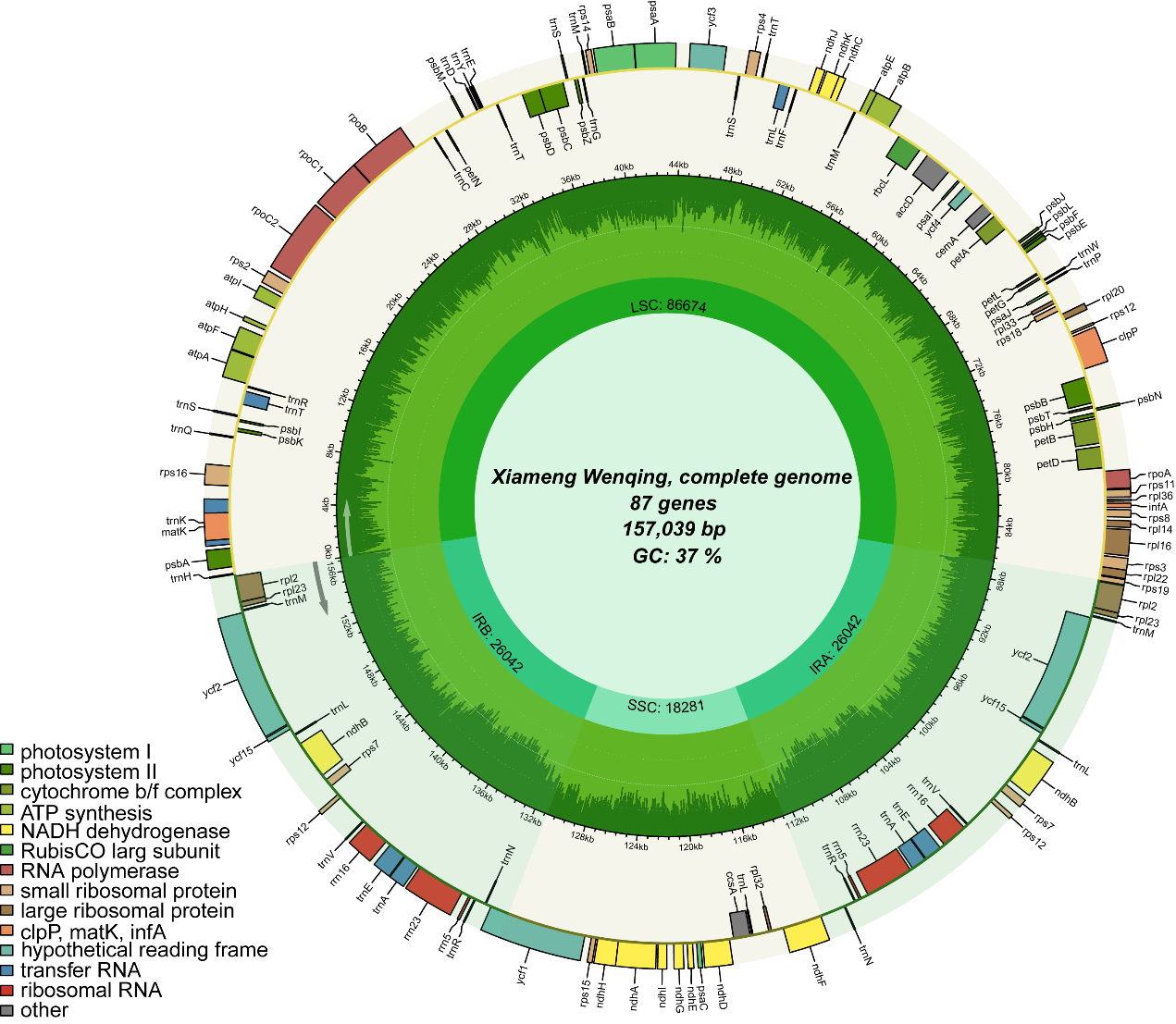


Figure S6 Circular map showing the complete chloroplast (cp) genome of *Camellia japonica* ‘Xiameng Wenqing’. This map includes key features of a cp genome, including the large single-copy (LSC) region, small single-copy (SSC) region, and a pair of inverted repeat (IR) regions.


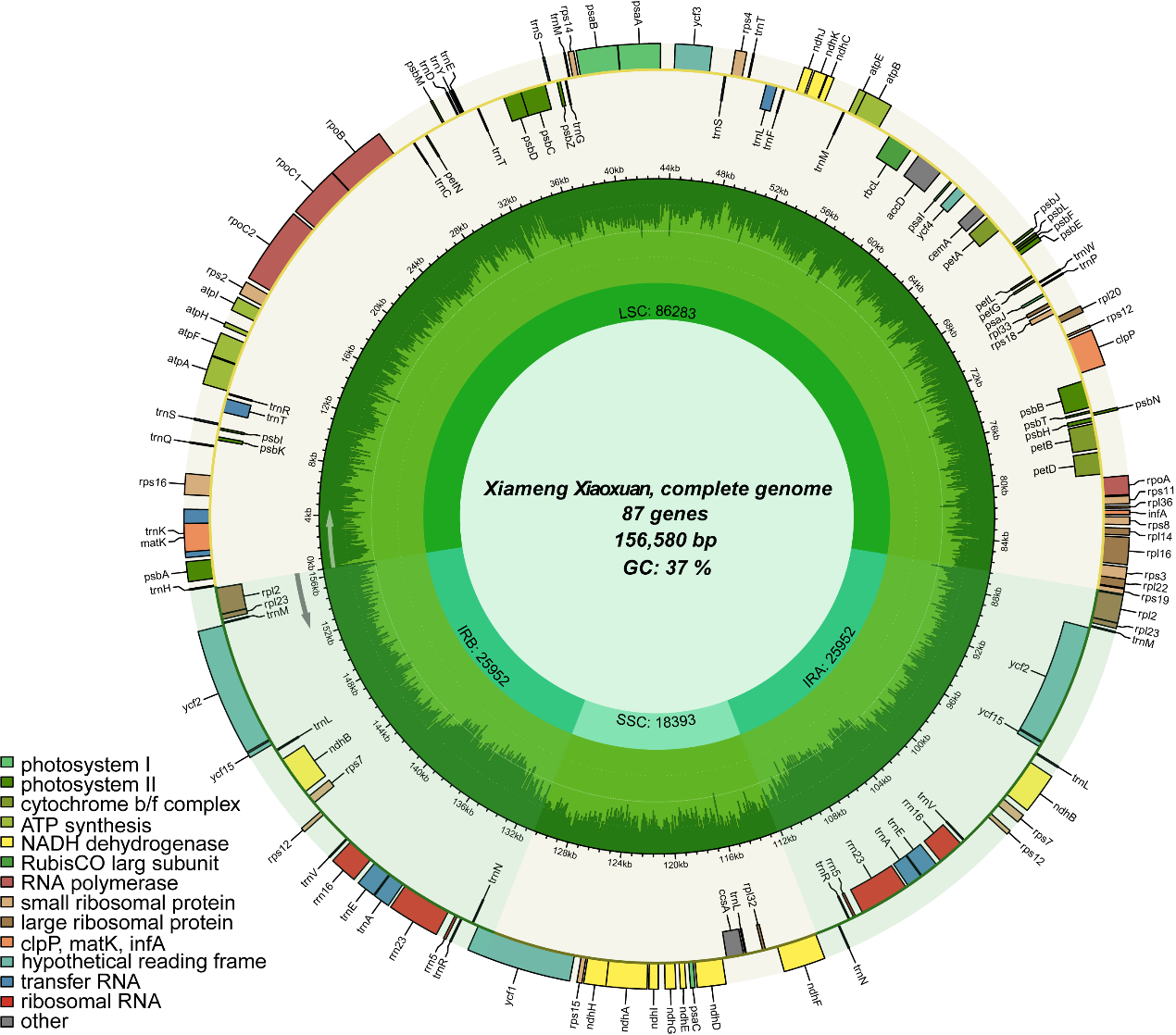


Figure S7 Circular map showing the complete chloroplast (cp) genome of *Camellia japonica* ‘Xiameng Xiaoxuan’. This map includes key features of a cp genome, including the large single-copy (LSC) region, small single-copy (SSC) region, and a pair of inverted repeat (IR) regions.


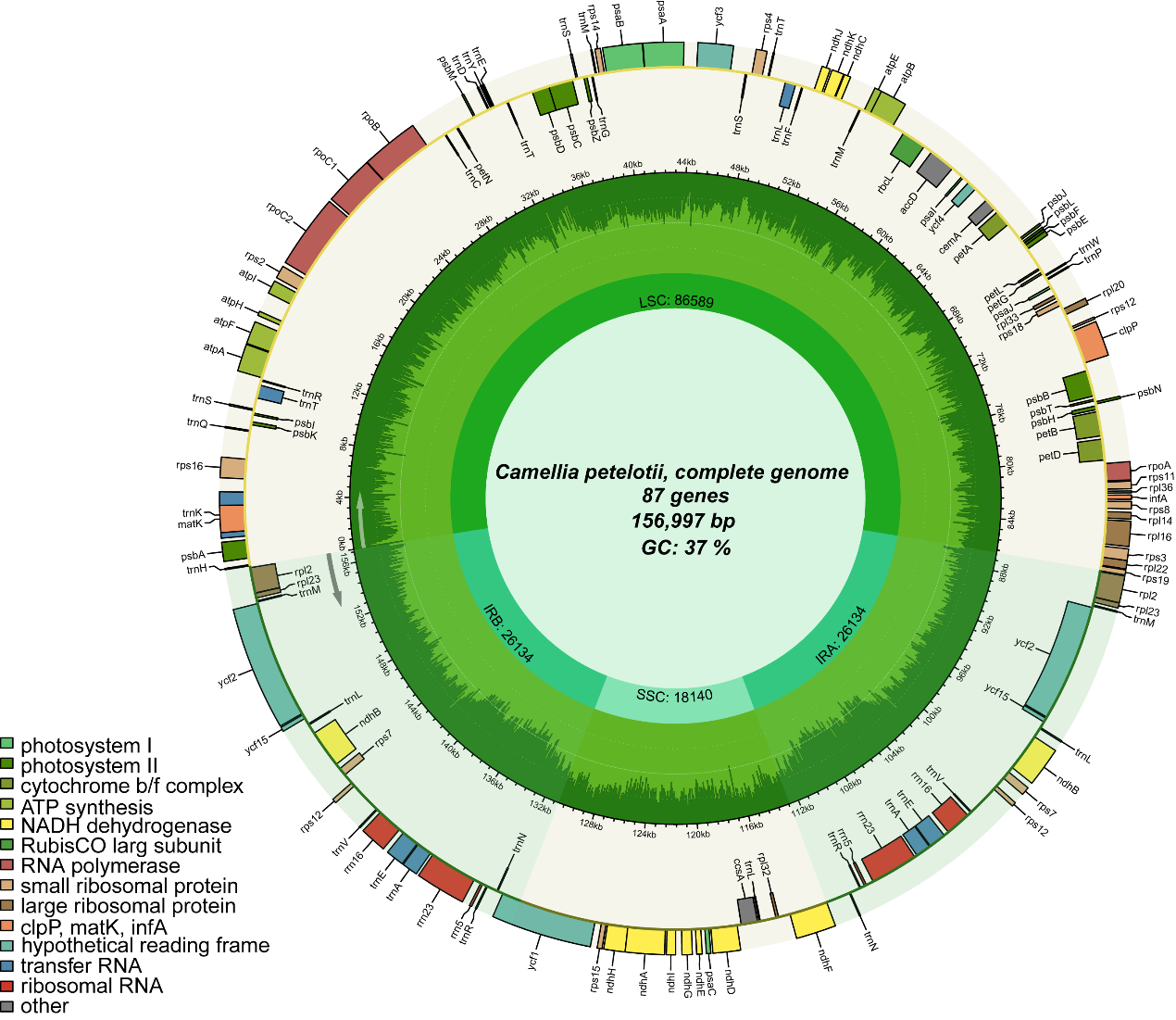


Figure S8 Circular map showing the complete chloroplast (cp) genome of *Camellia petelotii*. This map includes key features of a cp genome, including the large single-copy (LSC) region, small single-copy (SSC) region, and a pair of inverted repeat (IR) regions.


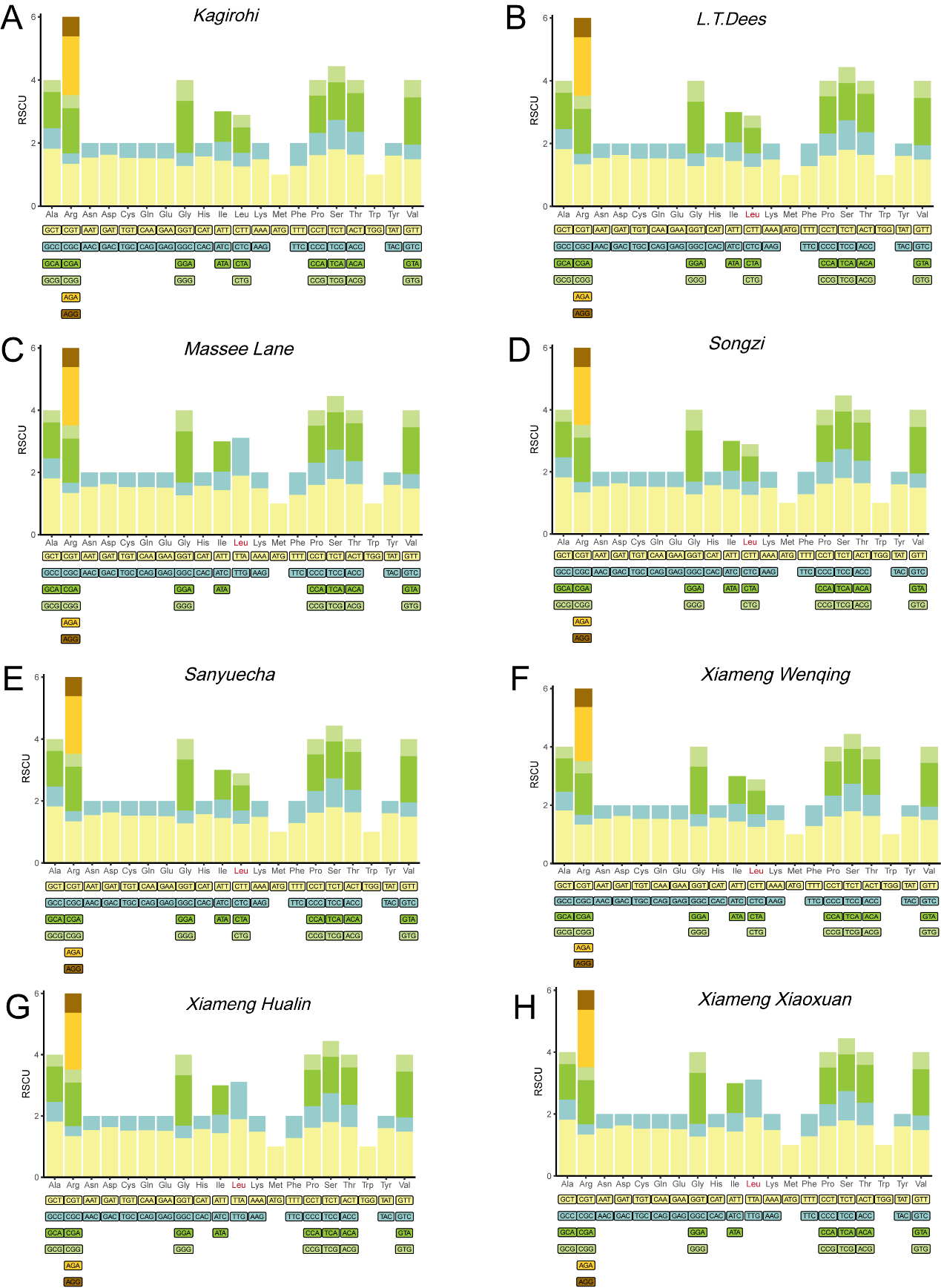


Figure S9 Codon usage analysis of coding sequences (CDSs) from the cp genomes of *Kagirohi, L.T.Dees, Massee Lane, Songzi, Sanyuecha, Xiameng Wenqing, Xiameng Hualin, and Xiameng Xiaoxuan.*
